# Ethical Considerations of Social Robots for Supporting Mental Health and Wellbeing in Older Adults in Long-Term Care

**DOI:** 10.1093/geroni/igaf122.2798

**Published:** 2025-12-31

**Authors:** Lillian Hung, Yong Zhao, Hadil Adfares, Parsa Shafiekhani

**Affiliations:** University of British Columbia, Vancouver, British Columbia, Canada; The University of British Columbia, Vancouver, British Columbia, Canada; The University of British Columbia, Vancouver, British Columbia, Canada; The University of British Columbia, Vancouver, British Columbia, Canada

## Abstract

Social robots are increasingly becoming utilized to address mental health challenges in older adults, such as depression, anxiety, and loneliness. However, ethical concerns surrounding their use are insufficiently explored in empirical research. This paper examines the ethical challenges and mitigation strategies for implementing social robots in long-term care settings. Drawing from insights gained over research across two Canadian studies involving Paro and Lovot, we highlight the critical role of an equity-focused approach to ensure the ethical use of social robots. We advocate for the respectful inclusion of the voices and desires of marginalized groups, such as older adults with dementia. Key ethical issues discussed include inequitable access, consent, substitution of human care, and concerns about infantilization. Our empirical work offers practical strategies to navigate these challenges, aiming to ensure that social robots promote mental health and well-being in an ethically responsible manner for older adults living in long-term care.

